# Uncertainty-Aware Multimodal Trajectory Prediction via a Single Inference from a Single Model

**DOI:** 10.3390/s25010217

**Published:** 2025-01-02

**Authors:** Ho Suk, Shiho Kim

**Affiliations:** 1 Seamless Trans-X Lab (STL), School of Integrated Technology, Yonsei University, Incheon 21983, Republic of Korea; sukho93@yonsei.ac.kr; 2 BK21 Graduate Program in Intelligent Semiconductor Technology, Yonsei University, Incheon 21983, Republic of Korea

**Keywords:** uncertainty quantification, single forward pass, multimodal trajectory prediction, autonomous driving, advanced driver assistance systems, edge platform

## Abstract

In the domain of autonomous driving, trajectory prediction plays a pivotal role in ensuring the safety and reliability of autonomous systems, especially when navigating complex environments. Unfortunately, trajectory prediction suffers from uncertainty problems due to the randomness inherent in the driving environment, but uncertainty quantification in trajectory prediction is not widely addressed, and most studies rely on deep ensembles methods. This study presents a novel uncertainty-aware multimodal trajectory prediction (UAMTP) model that quantifies aleatoric and epistemic uncertainties through a single forward inference. Our approach employs deterministic single forward pass methods, optimizing computational efficiency while retaining robust prediction accuracy. By decomposing trajectory prediction into velocity and yaw components and quantifying uncertainty in both, the UAMTP model generates multimodal predictions that account for environmental randomness and intention ambiguity. Evaluation on datasets collected by CARLA simulator demonstrates that our model not only outperforms Deep Ensembles-based multimodal trajectory prediction method in terms of accuracy such as minFDE and miss rate metrics but also offers enhanced time to react for collision avoidance scenarios. This research marks a step forward in integrating efficient uncertainty quantification into multimodal trajectory prediction tasks within resource-constrained autonomous driving platforms.

## 1. Introduction

Since the great success of the deep neural network with its impressive performance in several challenges, deep learning has been widely adopted in various applications in the past decade. Deep learning, with its continued technological advancements, has become a common option for driving automation, achieving a significant level of functional implementation in tasks such as perception, prediction, decision-making, and path planning that were difficult to model in detail using traditional methods. As a result, today’s driving automation systems equipped with deep learning models have reached a level of performance that users can accept, such as being able to be engaged not only in highway but also in complex downtown driving environments.

However, the autonomous vehicles currently in service correspond to SAE J3016 Level 4. Those autonomous vehicles do not require a human driver to take over driving responsibility but have not yet reached full automation equivalent to SAE J3016 Level 5 and have a limited ODD (Operational Design Domain) that can only operate in a specific predefined local area. This is because the safety of autonomous vehicles cannot yet be guaranteed in all driving situations.

Safety is the most important factor in automobiles, and to ensure safety, the automotive industry complies with the automotive safety standard ISO 21448 (Available online: https://www.iso.org/standard/77490.html, accessed on 8 November 2024) in the developmental process for driving automation systems. The goal of ISO 21448 is to ensure the SOTIF (Safety Of The Intended Functionality) of the systems that make up the automobile. ISO 21448 briefly introduces that aleatoric uncertainty and epistemic uncertainty should be considered during the development process of machine learning-based components. Deep learning models suffer from uncertainty, and driving automation systems based on them also face various uncertainty problems in real-world driving situations. In autonomous vehicles, uncertainty is a potentially hazardous issue and needs to be addressed more actively. If the uncertainty problem is not considered, the safety of the driving automation system cannot be guaranteed in all driving situations. In order to solve the uncertainty problem of deep learning models installed in autonomous vehicles, we must first find out which data and driving situations cause the output of the deep learning model to become uncertain.

Trajectory prediction for surrounding vehicles serves as a crucial intermediary task linking the perception subsystem, which extracts information from sensor data, with the planning subsystem, which is responsible for the decision-making processes of an autonomous vehicle. The predicted trajectory provides direct rationale for the planning of autonomous vehicles. In the trajectory prediction task, uncertain prediction can lead to incorrect decision-making, which may cause dangerous situations such as accidents. To this safety-critical task of trajectory prediction for surrounding vehicles, many existing studies utilize Deep Ensembles to quantify the uncertainty of predicted trajectories. While this approach has proven effective in various scenarios, its high computational overhead poses significant challenges in edge platforms. Deep Ensembles, which require multiple models to be trained and inferred, require high computing power and memory volume, as well as long calculation times. Those multiple models may not be feasible in the autonomous vehicle environment where hardware availability is limited. This infeasibility is also the same for Monte Carlo Dropout, which requires at least tens of inferences for sampling. In general, the perception subsystem of autonomous vehicles requires many sensors and heavy deep learning models for object detection and scene segmentation tasks to accurately recognize the surrounding environment. In order to reduce the load on the entire autonomous driving system, it is better for trajectory prediction to not have a large computational burden.

We quantify the uncertainty of predicted trajectories using a single inference from a single-model approach, which is suitable for limited execution constraints. Our approach shows comparable or better capabilities than state-of-the-art methods for quantifying uncertainty, despite its relatively low computational demands. Our uncertainty quantification approach is based on some state-of-the-art deterministic single forward pass studies. We decompose the trajectory prediction problem into a problem of predicting velocity and yaw, which allows us to quantify uncertainty for velocity and yaw separately. It means that we can obtain longitudinal uncertainty corresponding to velocity and lateral uncertainty corresponding to yaw separately. Moreover, for each longitudinal and lateral uncertainty, we can disentangle them into aleatoric uncertainty and epistemic uncertainty.

By incorporating longitudinal and lateral uncertainty into the trajectory calculation, we perform uncertainty-aware trajectory prediction. At moments with high aleatoric uncertainty, by considering both high-probability velocities and yaws, we predict multimodal trajectories which extract trajectories in a hydra-head-like shape. To the best of our knowledge, this is the first approach to multimodal trajectory prediction that leverages quantified uncertainty. In situations where the driving environment contains inherent randomness, this intuitive method of considering all likely predictions turns out to be effective, as evaluated by the minFDE and miss rate, which are default metrics for multimodal trajectory prediction. When modeling the intersection area between the trajectories of two vehicles as a risk, the multimodal trajectory reflecting uncertainty can allow the ego vehicle to have a longer reaction time for a surrounding vehicle whose predictions are uncertain, which can lead to safe driving to avoid collisions.

In summary, the contributions of our work are as follows:

- Demonstrating the feasibility of adopting a single inference from a single-model approach based on deterministic single forward pass methods for uncertainty quantification in a trajectory prediction task on edge platforms of autonomous vehicles with limited resources and computing power;
- Proposing the uncertainty-aware multimodal trajectory prediction (UAMTP) leveraging uncertainty through disentangled quantification of aleatoric and epistemic uncertainties in the longitudinal and lateral direction of vehicles;
- Demonstrating that uncertainty-aware multimodal trajectory prediction (UAMTP) can improve the safety of autonomous vehicles in driving scenarios with inherent uncertainties.

The remainder of this paper is organized as follows: Section 2 provides background knowledge on uncertainty quantification and describes prior works that leverage uncertainty in trajectory prediction tasks. Section 3 details how to decompose the trajectory prediction problem into the problem of predicting velocity and yaw, quantify longitudinal and lateral uncertainties, and perform uncertainty-aware multimodal trajectory prediction. Section 4 reports quantitative and qualitative experimental results and analyses to evaluate our uncertainty quantification and uncertainty-aware multimodal trajectory prediction approaches. Section 5 concludes this paper with a discussion of the results, limitations, and future work.

## 2. Background and Related Works

### 2.1. Types of Uncertainty

A probability vector output by the classifier model, along with its prediction, is not an appropriate metric for measuring confidence when the input data deviate from the distribution of the training data [1]. This is because the probabilities of the model may not accurately reflect uncertainty under such conditions, potentially resulting in incorrect confidence estimates. In deep learning models, it is highly recommended to quantify the uncertainty about the output to ensure a higher level of safety and reliability in the model’s predictions. Uncertainty is especially important in domains such as autonomous vehicles where human lives are involved. Uncertainty about the output of deep learning models can be categorized into aleatoric uncertainty and epistemic uncertainty, as follows.

#### 2.1.1. Aleatoric Uncertainty

Aleatoric uncertainty is a metric that quantifies the ambiguity of data resulting from sensor noise and the randomness of the driving environment. Since noise and randomness are inherent in the data to a certain extent, aleatoric uncertainty cannot be relieved by increasing the amount of training data. The most obvious way to reduce aleatoric uncertainty is to use sensors with more robust measurement specifications, so it is often considered a problem outside of deep learning.

#### 2.1.2. Epistemic Uncertainty

Epistemic uncertainty is a metric that quantifies the lack of knowledge required for a model to explain data. Unlike aleatoric uncertainty, epistemic uncertainty can be reduced by collecting more diverse and representative data or by improving the model architecture. Epistemic uncertainty is significant in safety-critical applications such as autonomous driving systems, as it can detect situations where the model is uncertain about its predictions due to unfamiliar input conditions [2].

### 2.2. Methods for Uncertainty Quantification

Methods for uncertainty quantification can be broadly categorized into five main approaches: the Bayesian model, Monte Carlo Dropout, Deep Ensembles, Deep Evidential Regression, and deterministic single forward pass model. These methods provide diverse strategies for uncertainty quantification, each with its own advantages and limitations, as described below.

#### 2.2.1. Bayesian Model

The Bayesian model is based on Bayesian statistics, sets model parameters to random variables with probability distributions, and outputs probability distributions [3]. In the Bayesian model and its approximation methods, the variance or entropy of the prediction distribution represents the uncertainty of the model.

However, Bayesian models, due to their inherently complex and non-standard structure, often face limitations in leveraging existing state-of-the-art models and techniques. This structural complexity can hinder the integration of advanced architectures and optimization methods commonly used in deep learning, thereby constraining their applicability and scalability in practice. Furthermore, because the posterior distribution calculation is computationally intensive, it has the limitation of being difficult to scale to huge datasets or large models.

To alleviate this limitation, the variational inference method was proposed, which replaces the intractable posterior with a simpler task of optimizing the parameters of a variational distribution [4,5,6]. Monte Carlo Dropout, which will be described next, is a practical approximation technique leveraging dropout regularization to simulate a posterior distribution.

#### 2.2.2. Monte Carlo Dropout

Monte Carlo Dropout is a method for approximating Bayesian inference. It quantifies uncertainty by sampling dozens of different subnetworks and approximating the posterior distribution, making it relatively more practical than the Bayesian model [7]. Moreover, it has the advantage of being able to easily apply by simply adding the dropout mechanism to a layer in the existing deep neural network architecture.

However, since it requires multiple forward passes through the network during inference, it still has an increased computational cost compared to the standard model. An adaptive Monte Carlo Dropout method has been proposed to reduce the number of forward passes dynamically [8]. Nevertheless, Monte Carlo Dropout does not fundamentally overcome the limitation that approximation methods sacrifice robustness in uncertainty estimation [9]. Another disadvantage is that the performance of Monte Carlo Dropout is sensitive to the choice of a hyperparameter called the dropout rate, so extensive tuning is required [10,11].

#### 2.2.3. Deep Ensembles

Deep Ensembles are based on frequentist statistics rather than Bayesian statistics and aggregate the outputs of multiple independently trained models [12]. In Deep Ensembles, uncertainty is the variance or entropy of the outputs of all models that makes up the ensemble. The Deep Ensembles method is an intuitive yet powerful method of uncertainty quantification.

Although several comparative studies have shown that Deep Ensembles outperform Bayesian approximation [13,14], it is resource-intensive and computationally intensive method, because it requires training multiple models that make up the ensembles and running multiple models during the inference process. In general, increasing the number of networks that make up an ensemble improves the accuracy of prediction and the performance of uncertainty quantification [15,16]. This means that since a certain number of networks are essential for Deep Ensembles, the computational cost is a problem that is difficult to completely solve.

#### 2.2.4. Deep Evidential Regression

Deep Evidential Regression is a method to measure uncertainty for regression tasks using only a single forward pass model. This method estimates the mean and variance of the prediction target using the NIG (Normal Inverse-Gamma) distribution, thereby simultaneously learning aleatoric uncertainty and epistemic uncertainty [17].

However, the effect of the NIG prior with four parameters on the performance of uncertainty quantification is complicated. It is pointed out that the unique activation function that makes the NIG prior parameter non-negative can cause evidence contraction and interfere with optimization, and that the NIG distribution does not actually disentangle aleatoric uncertainty and epistemic uncertainty [18]. Due to theoretical defects, Deep Evidential Regression is a heuristic, not an uncertainty quantification [19].

#### 2.2.5. Deterministic Single Forward Pass

Deterministic single forward pass methods aim to quantify uncertainty in deep learning models without the need for multiple stochastic forward passes or ensemble predictions. A single forward pass model that requires only a single inference makes it computationally efficient compared to Bayesian models, Monte Carlo Dropout, and Deep Ensembles. It shows state-of-the-art uncertainty quantification performance while being efficient. In this approach, several methods have been proposed, but they only have in common that they utilize a deterministic single forward pass model. Each defines uncertainty in its own way and quantifies it through a single inference.

DUQ utilizes a model based on radial basis function networks, which compute distances to input data and generate outputs based on those distances [20]. It quantifies uncertainty with kernel distances based on Mahalanobis distances to determine out-of-distribution data. The greater the distance between input data and the feature space of the class, the higher the uncertainty that is given.

SNGP regularizes the feature space through spectral normalization (SN) and combines a Gaussian process (GP) in the last layer of the network to output the mean and variance of the predictions [21]. The Gaussian process calculates the prediction variance based on the distance between the input data and the learned data, which presents the model with higher uncertainty in areas where it has not learned.

DUE also quantifies uncertainty by utilizing the Gaussian Process [22]. However, DUQ, SNGP, and DUE have the limitation that they cannot disentangle aleatoric uncertainty and epistemic uncertainty. DDU can quantify aleatoric uncertainty with softmax entropy and epistemic uncertainty with GMM density, respectively, while showing state-of-the-art uncertainty quantification performance [23]. By modeling the feature space for each class with a GMM, it obtains the probability density that indicates how well the data belong to the corresponding class.

### 2.3. Trajectory Prediction in Autonomous Driving

The autonomous driving system consists of three subsystems: perception, planning, and actuation. The perception subsystem gathers and processes sensor data to detect surroundings of the ego vehicle. The planning subsystem analyzes the processed information to make decisions about the path and actions of the ego vehicle. The actuation subsystem executes the planned maneuvers by controlling the ego vehicle. Trajectory prediction for surrounding vehicles functions as a crucial intermediary task that bridges the perception subsystem and the planning subsystem. The predicted trajectories serve as foundational inputs, offering direct justification for the planning and decision-making of autonomous vehicles. Here, we review recent works on trajectory prediction with uncertainty quantification of vehicles and recent works on multimodal trajectory prediction.

#### 2.3.1. Uncertainty Quantification in Trajectory Prediction

In recent years, uncertainty quantification has emerged as one of the fast-growing research fields, as it has attracted attention for its importance in contributing to the robustness and safety of deep learning. This trend is also prominent in the field of safety-critical autonomous driving, where many studies have been published on applying uncertainty quantification to trajectory prediction. The main approach used in this context is the Deep Ensemble method, which has been widely adopted due to its intuitiveness and high accuracy.

Tang et al. [24] construct an LSTM-based motion prediction model using the Deep Ensembles method to quantify aleatoric uncertainty and epistemic uncertainty. Their UADMF framework uses predictions and uncertainties to form an uncertainty-aware potential field and performs decision-making using the potential field. Shao et al. [25] propose the GRIP+++ model, which constructs a graph convolution-based trajectory prediction model using the Deep Ensembles method. Their method utilizes quantified uncertainty to detect failure in motion prediction. Li et al. [26] propose UQnet, which quantifies aleatoric uncertainty and epistemic uncertainty in trajectory prediction by constructing a mesh-grid 2D histogram-based model with Deep Ensembles.

There are also studies that apply Deep Ensembles to perception tasks that provide processed information as input to a trajectory prediction model [27]. Peng et al. [28] propose the Self-Surveillance and Self-Adaption System that includes a module for quantifying SOTIF entropy by configuring the YOLOv5 model with the Deep Ensembles method. The entropy, which measures both perception algorithm risk and collision risk, is used for the decision-making process using potential fields to drive the vehicle in the way of lowering the entropy.

There are also studies where Deep Ensembles have been applied to RL for autonomous driving tasks other than trajectory prediction. Hoel et al. [29] introduce EQN, which quantifies aleatoric uncertainty and epistemic uncertainty by applying Deep Ensembles to distributed RL while performing autonomous driving, thereby achieving safer driving. Yang et al. [30] propose the RDMF framework that constructs Deep Deterministic Policy Gradient (DDPG) RL models as Deep Ensembles. RDMF quantifies model uncertainty to identify unseen scenarios and determines whether the RL policy is safe.

Adopting Deep Ensembles for uncertainty quantification in autonomous driving is a popular option. However, as explained in Section 2.2.3, Deep Ensembles require multiple networks for accurate uncertainty quantification. This means that compared to a general model that does not form an ensemble, they requires several times more memory proportional to the number of networks and longer inference time [26]. Deep Ensembles may not be suitable for edge platforms for autonomous vehicles where hardware availability is limited due to price and power consumption considerations.

#### 2.3.2. Multimodal Trajectory Prediction

Trajectory prediction is inherently a multimodal problem because there are multiple possible future trajectories that vehicles or pedestrians can take from a single state. Due to various factors such as road structure or agent intentions, it is impossible to predict a correct single deterministic trajectory, forcing the model to make uncertain predictions. Other contextual information can be used to infer the agent’s style or intention, but it has limitations [31]. Estimating driving style or driver intention requires looking at a much longer driving history than a trajectory prediction task. It is difficult to determine style or intention from a split second of movement. Tracking long-term history also requires incorporating object tracking tasks, as it must also respond to occlusion and reappearance of the vehicle in a real-world driving environment. There is also the problem that input for understanding driving intention cannot always be provided in the real world. For example, it is possible to estimate the direction in which a car will turn by looking at its turn signal. Unfortunately, autonomous vehicles will encounter human drivers who do not use their turn signal on real-world roads.

Combining driving style and driver intention recognition into the trajectory prediction task can help improve prediction accuracy in complex situations. Nevertheless, such an integrated framework is not easy to fully implement and may introduce another source of uncertainty from the new task. Unless we reach the era of fully connected vehicles, where future trajectories are communicated to other vehicles, the trajectory prediction tasks have to be performed in a multimodal way while considering the uncertainty issue. Performing multimodal trajectory prediction is necessary to provide rich information that leads to safer decision-making in autonomous driving systems.

Various approaches have been published to solve the multimodal trajectory prediction problem. Cui et al. [32] use a deep convolutional network to predict multiple possible trajectories and estimate the probability of each trajectory. Deo et al. [33] perform similar work with an LSTM-based encoder–decoder architecture, emphasizing temporal dependencies in trajectory sequences. Their method leverages the sequential nature of vehicle movements, ensuring more coherent trajectory predictions over time. In another study, Deo et al. [34] utilize an encoder with a graph neural network (GNN) architecture, enabling the model to capture relational interactions among multiple agents in a traffic environment. This GNN-based method has been particularly effective in scenarios involving dense traffic, where the interactions among vehicles significantly influence trajectory outcomes. Liu et al. [35] and Nayakanti et al. [36] use an encoder–decoder transformer network to solve the multimodal trajectory prediction problem. The transformer-based approach enhances the model’s ability to capture long-range dependencies and interactions, offering robust performance in predicting trajectories under complex conditions. Phan-Minh et al. [37] and Sun et al. [38] approach the multimodal trajectory prediction task as a classification work. Phan-Minh et al. propose CoverNet, which generates a set of trajectories for classification. Sun et al. propose PCCSNet, which applies clustering for classification. Each of these approaches contributes uniquely to addressing the challenges of multimodal trajectory prediction. While convolutional and recurrent neural networks focus on capturing spatial and temporal features, graph neural networks and transformers excel in modeling interactions and long-range dependencies. Classification-based methods, on the other hand, offer computational efficiency by reducing the complexity of trajectory generation.

Deep Ensembles are a powerful method for handling uncertainty and have been widely adopted for providing robust and reliable multimodal trajectory predictions across diverse input conditions. Lafage et al. [39] introduce a novel approach called Hierarchical Light Transformer Ensembles, which combines Deep Ensembles with Transformer-based models to improve the performance of multimodal trajectory forecasting. This study utilizes the hierarchical structure of Transformers to process input data at multiple resolutions, enabling each ensemble model to generate more precise and detailed trajectories. Nayak et al. [40] leverage Deep Ensembles to address trajectory forecasting under sensing uncertainty. Their approach involves independent predictions by ensemble models based on varied data sampling, allowing the calculation of the mean and variance of the predictions to effectively quantify model uncertainty. However, high computational cost and hardware requirements of Deep Ensembles pose challenges for real-time deployment in autonomous vehicles, emphasizing the need for alternative methods better suited to resource-constrained platforms.

## 3. Methods

**Figure 1:**
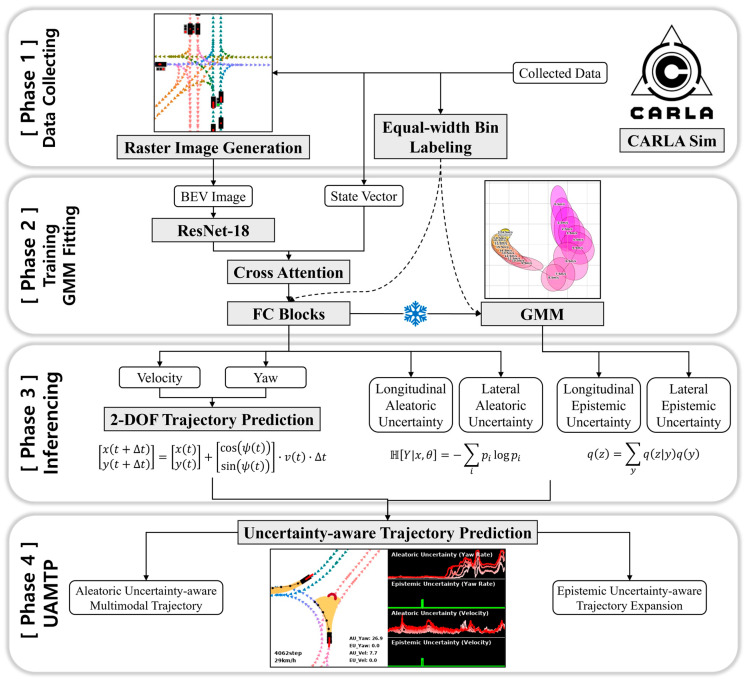
Workflow overview of proposed uncertainty-aware multimodal trajectory prediction (UAMTP) method. Phase 1 includes the process of data collecting and preprocessing covered in Section 3.1.3. Data collected from the CARLA simulator are preprocessed to generate BEV raster images and labeled for velocity and yaw prediction using equal-width binning. Phase 2 includes the training process covered throughout Section 3.1. Our model, consisting of ResNet-18, cross attention, and fully connected blocks, is trained to predict the velocity and yaw of vehicles. Phase 3 includes the process of 2-DOF trajectory prediction using predicted velocity and yaw covered in Section 3.1.2 and the process of quantifying the aleatoric uncertainty, defined as softmax entropy, and the epistemic uncertainty, defined as the feature likelihood from the GMM, covered in Section 3.2.3. Phase 4 covered in Section 3.3 includes the process of predicting the multimodal trajectories by reflecting the quantified aleatoric uncertainty and the process of expanding the predicted trajectory by reflecting the quantified epistemic uncertainty.

In Section 3, first, we introduce our approach to predict trajectories using an interpolated Euler method in a 2-DOF (Degree of Freedom) model after predicting the velocity and yaw of vehicles and describe our dataset and deep neural network-based model for it. Second, we explain our uncertainty quantification methodology for the trajectory prediction task based on a deterministic uncertainty quantification that requires only a single forward pass. Third, we explain in detail, with equations, how to interpret aleatoric and epistemic uncertainty in the trajectory prediction task and elaborate our multimodal trajectory prediction method using aleatoric uncertainty and our trajectory expansion method using epistemic uncertainty. The experimental results demonstrating the proposed methods are presented in each subsection of Section 4 corresponding to each subsection of Section 3. Figure 1 shows the workflow overview of the proposed uncertainty-aware multimodal trajectory prediction (UAMTP) method.

### 3.1. Decomposition of Trajectory Prediction Task

#### 3.1.1. Decomposition into Velocity and Yaw Prediction Task

Trajectory prediction is mainly treated as a regression task since it predicts continuous spatial coordinates over time. On the one hand, there are studies that approach trajectory prediction as a classification task [37,41]. They usually adopt a method of selecting the most appropriate one among various types of trajectory shapes. In this study, instead of predicting the trajectory directly by regression or classification on a trajectory set, we decompose the trajectory prediction task into a classification task of predicting velocity, the first derivate of position with respect to time, and predicting yaw.

To define our prediction task as a classification task, we apply discretization in the preprocessing process, which divides the continuous values of velocity and yaw into multiple bins and converts them into discrete labels, which are representative values of each bin. There are traditional but still adopted supervised methods for discretization that aim to classify continuous values [42,43], as well as recently devised advanced methods [44,45]. Finding the most effective discretization method for a specific application is still a challenging problem [46]. In our work, we utilize equal-width binning, an unsupervised method that does not require complex hyperparameters. Velocity is binned at 1 m/s intervals. The label of the 0–1 m/s bin is 0, and the representative value of this bin is the median, 0.5 m/s. Continuing, the label of the 1–2 m/s bin is 1, and the representative value is 1.5 m/s. Likewise, the label of the 24–25 m/s bin is 24, and the representative value is 24.5. In this way, velocity is divided into a total of 25 classes.

Yaw is binned at 5-degree intervals. The method of setting the labels and representative values is the same as for velocity. Yaw is divided into a total of 71 classes. The label of the −177.5–172.5 degree bin is −35, and the representative value of this bin is the median of the range, −175 deg. The label of the −172.5 to −167.5-degree bin is −34, with a representative value of −170 degrees. Similarly, the label of the −7.5 to −2.5-degree bin is −1, and its representative value is −5 degrees. The −2.5 to 2.5-degree bin is assigned a label of 0, with a representative value of 0 degrees. Continuing in the same manner, the label of the 2.5 to 7.5-degree bin is 1, with a representative value of 5 degrees. The 167.5 to 172.5-degree bin is labeled 34, with a representative value of 170 degrees, while the 172.5 to 177.5-degree bin is labeled 35, with a representative value of 175 degrees.

By converting continuous values into representative values within a bin, we can expect a smoothing effect that reduces the influence of noise existing in continuous values [47]. In the structure where the median value of each bin is the representative value, we convert the predicted classes of velocity and yaw into values and ultimately use them to predict trajectories. The trajectory prediction method is described in Section 3.1.2.

Rather than predicting yaw directly, our model predicts the future relative yaw, that is, the difference between the current yaw and the future yaw, and then calculates the future yaw in postprocessing. For example, if the current yaw of a vehicle is 30 degrees and the predictor model predicts that the yaw difference of the vehicle after 10 timesteps will have a class of 1, i.e., 5 degrees, then we predict that the vehicle will have a yaw of 30 + 5 = 35 degrees after 10 timesteps. The reason why yaw is not chosen as a direct prediction target is because yaw under the world coordinate system depends on the direction that roads face on the map. In other words, the yaw as a prediction target makes the model learn the direction of roads rather than the direction of the vehicle. Models trained to directly predict the yaw generalize poorly on maps that the model has not encountered during the training process.

#### 3.1.2. Trajectory Prediction Task

Based on the predicted velocity and yaw, an interpolated Euler method in a 2-DOF model is employed to predict trajectories of target vehicles. The 2-DOF model considers both the forward motion and rotational motion of a vehicle. The state vector $s\left(t\right)$ includes the position $\left(x,y\right)$ of a vehicle:

(1) $$s\left(t\right)=\left[\begin{array}{c} x\left(t\right)\\ y\left(t\right) \end{array}\right].$$

Through the equation below, the state vector $s\left(t\right)$ is updated at each timestep based on the predicted velocity v and yaw ψ, allowing for propagation of vehicle dynamics over discrete time intervals:

(2) $$\left[\begin{array}{c} x\left(t+\Delta t\right)\\ y\left(t+\Delta t\right) \end{array}\right]=\left[\begin{array}{c} x(t)\\ y(t) \end{array}\right]+\left[\begin{array}{c} \cos\left(\psi \left(t\right)\right)\\ \sin\left(\psi \left(t\right)\right) \end{array}\right]\cdot v\left(t\right)\cdot \Delta t.$$

To account for changes in the predicted velocity and yaw over time, our model employs linear interpolation. The predicted sequential velocity and yaw at specified future timesteps (e.g., after 10, 20, 30, and 40 timesteps in a CARLA simulator running at 20 Hz) provide discrete data that represent the expected dynamic behavior of a vehicle at specific future moments. To obtain continuous trajectory predictions across the entire prediction horizon, the velocity and yaw between the predicted points are interpolated linearly. The model assumes that a vehicle moves with constant yaw and velocity over each time interval Δt. However, by making the time interval Δt sufficiently small, the model can approximate the constant acceleration and yaw rate assumption. There have been many studies using the constant yaw rate and velocity model or the constant yaw rate and acceleration model on trajectory prediction tasks, and it has been proven that they can make accurate predictions at a reasonable level [48,49,50,51,52]. The interpolated values are then used in the 2-DOF model to update the state vector $s\left(t\right)$ according to Equation (2). By utilizing the linear interpolation method, our model provides smooth and continuous predictions of the future trajectory of a vehicle while still ensuring computational efficiency.

#### 3.1.3. Dataset

**Figure 2:**
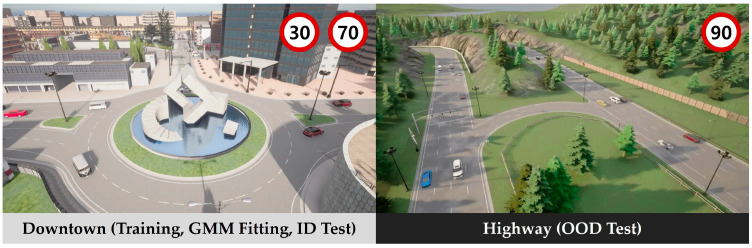
Two different driving environments were used for training and testing the model in this study. Town03 is a downtown environment with a low speed limit, and Town06 is a highway environment with a high speed limit.

To train our model, fit the GMM, and evaluate the model, we collected driving data using the CARLA simulator [53]. To create the training dataset and validation dataset used for model training and GMM fitting, we collected the driving dataset from the Town03 map, which is a downtown environment with multiple crossroads and one roundabout. Meanwhile, to create the OOD test dataset used to evaluate the ability of model detecting OOD (Out Of Distribution), we collected the driving dataset from the Town06 map, which is a highway environment with up to 6 lanes. Town03 and Town06 not only have different road networks but also different speed limits. For a model trained only in Town03, the unfamiliar road network and high-speed vehicles in Town06 are OOD cases. Figure 2 shows representative images of the Town03 and Town06 driving environments.

In each map, we collect a total of 501 driving sequence data points, and the 501 driving sequences consist of 167 driving sequences for each driving environment with light traffic (150 vehicles), moderate traffic (200 vehicles), and heavy traffic (250 vehicles), allowing the model to learn from various traffic volumes. In each driving sequence, vehicles are spawned at random points. Spawned vehicles continue to roam along the road toward randomly updated destinations. Data are collected at a rate of 20 Hz, and a single driving sequence is 5000 timesteps long, i.e., 250 s. At each timestep, a state vector is collected for each vehicle. Table 1 describes the attributes that make up the state vector and their units.

By integrating the state vectors of vehicles with the road information data of the map, a BEV (Bird’s Eye View) raster image is created that represents the surrounding context of each vehicle. The BEV raster image contains the positions and headings of the vehicles, the paths the vehicles have traveled for 10 timesteps (0.5 s), the lanes on the road that indicate the heading direction and virtual center line of each lane, and the status of the traffic light that each vehicle is affected by.

Considering the sensing range and field of view of autonomous vehicles, the BEV raster image shows only a certain range of areas from each vehicle. In the BEV raster image, the frontward visibility distance is limited to tens of meters, corresponding to the detection distance of the wide-angle camera, and the rearward visibility distance is limited to 12.5 m. In addition, the state vector history of vehicles is limited to the past 0.5 s. Of course, if we use a longer history, we can expect better trajectory prediction performance of the model by adopting LSTM. For example, Waymo’s dataset allows predicting trajectories using 1 s of past history [54,55]. However, in real-world driving situations, it is not easy to continuously track the vehicle due to occlusion of sight. A vehicle that suddenly pops out is potentially more dangerous to the ego vehicle than a vehicle that has been continuously tracked, and for safety, trajectory prediction must be performed accurately even for targets with such short histories. Therefore, in this study, we limited accessibility to only very close past state vectors.

In this study, we focus on uncertainty quantification and uncertainty-aware multimodal trajectory prediction tasks. Rather than providing raw sensor data as input, we assume that the perception task has been processed in advance. The state vector and BEV raster image are provided together as inputs to the model. During the training process of the model, the driving sequences are not input sequentially but are provided through random sampling. Among 501 driving sequences, 300 are randomly selected and used for training. In a total of 300 training epochs, one driving sequence is used for each epoch. From each driving sequence of 5000 timesteps long, 70 timestep samples are randomly selected, and from each timestep, 125 vehicle samples are randomly selected. That is, 8750 samples are selected for each epoch, and 2,625,000 samples are selected throughout the entire training process. However, since the driving environment where we train the model is a downtown area, there are many cases where vehicles are stopped due to traffic lights. Among the samples, nine out of ten vehicle samples that were stationary during 40 timesteps, which is the prediction horizon, starting from the past 10 timesteps are filtered out and not used in training. This filtering is intended to encourage learning about a variety of driving situations.

In the CARLA simulation environment, we can construct and test scenarios that rarely occur in reality. This flexibility allows us to construct an edge case for the collision risk situation due to uncertainty in the roundabout environment, which is utilized in the last experiment in Section 4.3. It is possible to reproduce our results via the data generation code on our Github repository, as specified in the Data Availability Statement Section.

#### 3.1.4. Model Architecture

Our model is a ResNet-based structure [56]. The model receives a BEV raster image with a size of 320 × 320 through a convolutional neural network. The convolutional blocks are stacked with sufficient depth so that the receptive field size of the lower convolutional layer is wider than the input image. In detail, the first convolutional layer using a 7 × 7 kernel and stride of 2 is followed by a batch normalization and a max pooling layer using a 3 × 3 kernel and a stride of 2. There are a total of 8 convolutional blocks after the first 7 × 7 convolutional layer. Each convolutional block consists of two convolutional layers with batch normalization and a single residual connection. The convolutional layers that make up the convolutional block use a 3 × 3 kernel and a stride of 1. After the final layer of convolutional blocks, GAP (Global Average Pooling) is applied to make the model robust to spatial information in the BEV raster image [57,58]. By using the feature obtained from the convolutional neural network as a query and the state vector input as a key and value, cross attention is performed to combine important information between two different inputs [59]. After that, the combined information is fed into fully connected blocks.

The fully connected blocks are stacked in four stages. Each fully connected block consists of two fully connected layers with a single residual connection. The first stage has one block. The blocks of the second stage are divided into two branches, one each for velocity and yaw. In the third and fourth stage, each block for velocity and yaw is divided into four branches. Ultimately, the model predicts velocities and yaws after the specified timesteps (e.g., after 10, 20, 30, and 40 timesteps in a 20 Hz system) from the prediction moment. By predicting velocity and yaw separately, the quantification of longitudinal uncertainty and lateral uncertainty can be disentangled.

As explained earlier, each block consists of two layers and a single residual connection. Residual connections and spectral normalization are applied to all convolutional and fully connected layers to regularize the feature space of the model well [21,23,60]. A well-regularized feature space is useful in uncertainty quantification methods that utilize a proxy of the feature space [23]. The parameters used to train the network are as follows. As mentioned in Section 3.1.3, the training epoch is 300. The learning rate is 0.05, and the learning rate decay is 0.5. The momentum is 0.8, and the weight decay is 0.0005.

For the safety of autonomous vehicles, various tasks including trajectory prediction must be performed in real time, and the entire process of the autonomous driving system must operate at a rate of tens of times per second [61,62]. In general, researchers and developers agree that a rate of 20 Hz or more is recommended heuristically. Since designing a model with complex structure may hinder fast operation, our model is structured to be concise but suitable for quantifying uncertainty. Although, our approach does not require additional unusual network architectures for uncertainty quantification; therefore, it can be extended as needed.

### 3.2. Uncertainty Quantification

#### 3.2.1. Uncertainty Quantification Methods

As explained in the introduction, autonomous driving systems based on deep learning models suffer from uncertainty issues, which pose a potential risk to autonomous vehicles. In particular, uncertain trajectory prediction can lead to incorrect decision-making by the system, which may cause accidents. In order to deal with uncertainty issues, it is first necessary to quantify the uncertainty from the predictions of a deep learning-based model. As a result of the uncertainty quantification, we can determine in which driving situations the model makes uncertain trajectory predictions.

However, adopting uncertainty quantification comes with tradeoffs in terms of additional memory and computation time. Computational overhead can be a sensitive issue for autonomous vehicles. Autonomous vehicles are edge platforms that do not use the cloud and instead mount hardware with limited resources and computing capabilities on the vehicle body. To ensure real-time responsiveness, autonomous driving systems do not rely on the cloud and demand their models to be efficient. As mentioned in Section 3.1.4, the system must operate at a rate of tens of times per second. To satisfy real-time processing, it is necessary to find a balance between computational burden and performance in uncertainty quantification methods.

In Section 2.2, we introduced five state-of-the-art methods for uncertainty quantification. Among them, we selected three methods with high applicability because they do not require unique architectures or learning systems and then applied them to the trajectory prediction task described in Section 3.1. The key specifications of these three state-of-the-art uncertainty quantification methods we chose for evaluation are described in Table 2. Our single inference from a single-model approach builds on several deterministic single forward pass studies that show state-of-the-art performance in uncertainty quantification [20,21,22,23,63]. These deterministic single forward pass models have been proven effective in not only toy problems for 2D classification benchmarks such as two moons but also image classification tasks. We extend the deterministic single forward pass model to the trajectory prediction task. The model architecture of the deterministic single forward pass method is the same as described in Section 3.1.4, because it does not require any modification to the deep neural network structure. However, to quantify epistemic uncertainty from a single general model with only a single inference, we need to obtain a proxy representing the feature space density after the model is trained. In this study, we choose a GMM as the proxy, which was proposed in DDU [23]. The details of the GMM are described in Section 3.2.2.

In the case of Monte Carlo Dropout, dropout is applied to all fully connected blocks except the last block that extracts the prediction. Dropout is not applied to the convolutional blocks, because it can harm the image understanding of the model. The dropout rate is set to 0.1, which can create differences between samples while minimizing the degradation of the prediction performance. Except for the application of dropout, the model is the same as the deterministic single forward pass method. The models that make up the Deep Ensembles are also the same as the models of the deterministic single forward pass method. The only difference is that each model is trained under different seed settings. The evaluation results of these three methods for uncertainty quantification in the trajectory prediction task are compared in Section 4.2.

As explained in Section 2.2.1, Bayesian neural networks, which apply probability distributions to parameters to directly compute the posterior distribution, are not commonly adopted architectures due to their limited scalability. Because the process of inferring the posterior distribution incurs a large computational burden, we excluded Bayesian models from our evaluation. Deep Evidential Regression may seem suitable for uncertainty quantification in trajectory prediction tasks that handle continuous data. However, as described in Section 2.2.4, this method of learning the parameters of the NIG distribution to quantify uncertainty utilizes peculiar activation functions and loss terms. The disadvantage of this unusual learning method is that it is less applicable to existing models and is difficult to optimize. Above all, this method is a heuristic that does not actually quantify uncertainty. Therefore, we did not include Deep Evidential Regression in our evaluation.

#### 3.2.2. Gaussian Mixture Model

The deterministic single forward pass method, unlike Deep Ensembles, uses only one model, and unlike Monte Carlo Dropout, only one sample is used for the inference phase. In order to quantify epistemic uncertainty under the single inference from a single general model condition, a proxy that represents the feature space of the trained model is required. The use of a proxy is a common practice in deterministic single forward pass methods [23,63]. As mentioned in Section 3.2.1, we utilize the GMM to represent the feature space density of our trained model [23].

After the training is complete, the validation dataset consisting of eight hundred thousand samples is fed into the frozen model to obtain GMMs by GDA (Gaussian Discriminant Analysis). Specifically, by feeding samples into the model to extract encoded information of the fully connected block just before the last layer that performs prediction, we can obtain the embedded features of our deep learning-based model. At the same time, we also prepare the labels of the samples. Using the features and corresponding labels of the samples, we find the mean and covariance of each class, and finally fit the GMM.

Since our model predicts velocity and yaw separately, we obtain separate GMMs for velocity and yaw. As explained in Section 3.1.1, we convert continuous values of velocity and yaw into classes by applying equal-width binning. The number of Gaussian distributions that make up the GMM corresponds to the number of classes. For example, there are a total of 25 classes representing velocity, so the GMM of velocity consists of 25 Gaussian distributions. The obtained GMMs represent the density of the feature space and are utilized to quantify the epistemic uncertainty by calculating the marginal likelihood of the feature in the inference phase. More details on how to quantify the uncertainty using GMMs are covered in Section 3.2.3.

#### 3.2.3. Uncertainty Quantification Equations

As covered in Section 2.1, uncertainties in deep learning models can be divided into aleatoric uncertainty and epistemic uncertainty. The formula for quantifying aleatoric uncertainty appears to be nearly identical for the three methods: deterministic single forward pass, Deep Ensembles, and Monte Carlo Dropout. Aleatoric uncertainty is the entropy of the softmax probability distribution [23,64]. For the deterministic single forward pass method, it is calculated for a prediction from a single inference, which is called softmax entropy. For Deep Ensembles and Monte Carlo Dropout, it is calculated for the mean of multiple predictions, which is called predictive entropy:

(3) $$H\left[Y|x,\theta \right]=-\sum_{i}p_{i}\log p_{i}.$$

Although there may be confusion due to the similarity in the name or the form of the equation, cross entropy, which measures the difference between the distribution of actual labels and the probability distribution predicted by the model, and softmax entropy, which measures aleatoric uncertainty, are different. The minimum value of softmax entropy is 0, which appears when the probability is concentrated in a specific class. The maximum value appears when the probability is equally distributed for each class, which is about 3.22 when there are 25 classes and about 4.28 when there are 71 classes.

For the deterministic single forward pass method in our experiments, epistemic uncertainty is the marginal likelihood of the feature under the feature space. Thanks to using a GMM as a proxy for the feature space of the prediction model, we can quantify epistemic uncertainty by disentangling it with aleatoric uncertainty [23,60]:

(4) $$q\left(z\right)=\sum_{y}q\left(z|y\right)q\left(y\right).$$

If the amount of data per class in the data distribution is equal, there is no need to consider the prior $q\left(y\right)$ when quantifying epistemic uncertainty. However, even if the data per class are not evenly distributed, the influence of the prior $q\left(y\right)$ in a high dimension with at least dozens of classes is negligible compared to the likelihood $q\left(z|y\right)$. In actual implementation, compute the log-probability of each class feature under the GMM first, and then sum these log-probabilities, which corresponds to the marginal likelihood in the log-space. By multiplying by a negative at the end, we can interpret that the higher the value, the higher the epistemic uncertainty:

(5) $$q\left(z\right)=\log\sum_{y}e^{\log\left(q\left(z|y\right)\right)}.$$

For Deep Ensembles and Monte Carlo Dropout, we adopt the variance of predictions from multiple models or samples as epistemic uncertainty. The more inconsistent the predictions for one input, the more likely it is that knowledge of the model is insufficient because that input was not sufficiently provided to the model in the training phase. Additionally, for Monte Carlo Dropout, we also adopt the mutual information obtained by subtracting the expected softmax entropy from the predictive entropy as another epistemic uncertainty.

As explained in Section 3.1.1, since our model predicts velocity and yaw separately, we can obtain longitudinal uncertainty by quantifying uncertainty for velocity prediction and lateral uncertainty by quantifying uncertainty for yaw prediction in the trajectory prediction task. In other words, we can obtain longitudinal aleatoric uncertainty, longitudinal epistemic uncertainty, lateral aleatoric uncertainty, and lateral epistemic uncertainty separately. Through this, our method can disentangle the uncertainties of the predicted trajectory into the longitudinal and lateral directions, making it suitable for uncertainty-aware multimodal trajectory prediction, which is described in Section 3.3.

### 3.3. Uncertainty-Aware Multimodal Trajectory Prediction (UAMTP)

#### 3.3.1. Analysis of Uncertainty in Trajectory Prediction

As explained in Section 2.3.2, since there are multiple future trajectories of a vehicle that can be predicted from one state, multimodal trajectory prediction is essential for autonomous driving systems. With a single deterministic trajectory, it is impossible to always predict correctly, for example, whether a surrounding vehicle in the adjacent lane will change lanes into my lane or stay in its own lane, or which direction a vehicle on the opposite side will proceed at a crossroads without traffic lights. At the current level of technology, it is impossible to fully understand the hidden context in the driving environments, such as the intention of other agents. Therefore, trajectory prediction is a problem that inherently involves environmental randomness.

Modeling a task where randomness exists as a probability prediction is inappropriate because the predicted probability depends on the training data. This is because it is extremely difficult to collect a complete driving dataset that can be generalized to all driving situations. In the presence of uncertainty due to randomness, it is a safe approach to assume that all possible cases can occur. To solve the multimodal trajectory prediction task, we account for the aleatoric uncertainty induced by randomness. In object detection and scene segmentation tasks, aleatoric uncertainty is mainly used to quantify the noise in the image caused by the sensor. Meanwhile, in the trajectory prediction task, aleatoric uncertainty is suitable for quantifying the randomness inherent in the driving environment rather than the noise of the data. When the quantified aleatoric uncertainty is high, our method considers multiple possible velocities and yaws and eventually predicts multimodal trajectories in the form of hydra heads. In other words, multimodal trajectory prediction is performed only when aleatoric uncertainty is high, and unimodal trajectory prediction is performed otherwise, thereby increasing computational efficiency on the edge platform of autonomous vehicles.

**Figure 3:**
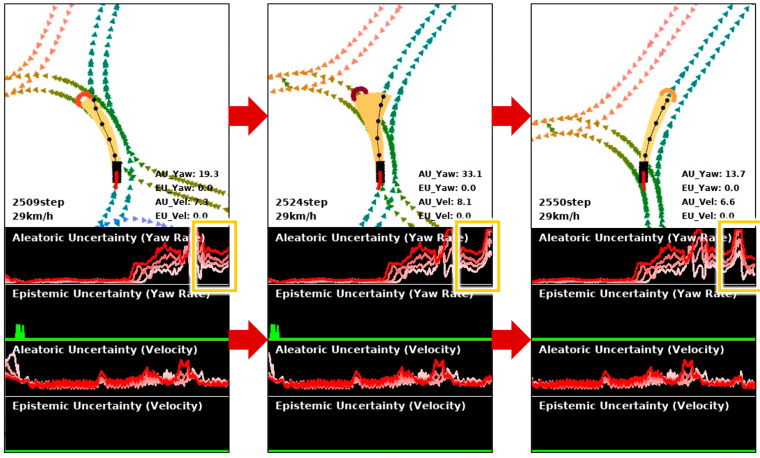
An example of qualitative results of the proposed uncertainty-aware multimodal trajectory prediction (UAMTP). A series of BEV raster images on the top shows the change in the predicted trajectory over timesteps, and a series of dynamic graphs on the bottom shows the change in the quantified uncertainty over timesteps. In the BEV raster image, the black rectangles represent the vehicle positions and headings, the red lines behind the vehicles represent the paths the vehicles have traveled for 10 timesteps (0.5 s), the black lines in front of the vehicles are the ground truth of the future trajectory, and the red polygon in front of the vehicle represents the predicted trajectory. Wedge-patterned lines indicate lanes containing the direction of travel. The dynamic graphs consist of four separate graphs. Each graph has four lines that represent the uncertainty of the predictions for after 10, 20, 30, and 40 timesteps.

Figure 3 shows an example of the proposed uncertainty-aware multimodal trajectory prediction in a roundabout driving environment. In the first figure on the left, a vehicle is circling around a roundabout, and the aleatoric uncertainty of yaw is not that high, so unimodal trajectory prediction is performed. In the middle figure, the vehicle reaches the two branches of the roundabout, where the trajectory is predicted multimodally due to increased aleatoric uncertainty of yaw caused by environmental randomness. In the right figure, as the vehicle starts to leave the roundabout, the aleatoric uncertainty decreases, and the trajectory prediction becomes unimodal again.

Meanwhile, in terms of epistemic uncertainty, high epistemic uncertainty means that the predictor model has not accumulated enough knowledge about the current driving situation during the training process. Predictions with high epistemic uncertainty are untrustworthy. And it is meaningless to perform multimodal trajectory prediction with unreliable predictions. As a conservative safety measure, we expand the area of the predicted trajectory of a vehicle with high epistemic uncertainty in its prediction. Trajectory expansion of vehicles with uncertainty leads the ego vehicle to make more careful decisions around those potentially hazardous vehicles. Since it is virtually impossible to find the optimal countermeasure for uncertain predictions after deploying the model, trajectory expansion is our last resort. The only solution is to improve the model by providing additional training for situations where knowledge is not sufficient.

#### 3.3.2. Uncertainty-Aware Multimodal Trajectory Prediction (UAMTP) Methods

In Section 3.3.1, we introduced our uncertainty-aware multimodal trajectory prediction (UAMTP) method. In this section, we explain our method by formulating it. Before going into specifics, we provide an overview of the equations discussed in this section to outline their roles within the proposed methodology.

Equations (6)–(24) describe the step-by-step process for obtaining aleatoric uncertainty-aware multimodal trajectories. Equations (6)–(12) explain how aleatoric uncertainty in velocity prediction is quantified and used to derive multimodal velocities from a longitudinal perspective, while Equations (13)–(18) outline the corresponding process for yaw prediction, focusing on deriving multimodal yaws from a lateral perspective. Following this, Equations (19)–(24) detail how the derived multimodal velocities and yaws are combined to obtain aleatoric uncertainty-aware multimodal trajectories. Lastly, Equations (25)–(28) shift focus to epistemic uncertainty, explaining the process for generating expanded trajectories based on quantified epistemic uncertainty.

Through the following series of equations, the number of the multimode in the longitudinal aspect is determined by reflecting the quantified aleatoric uncertainty of velocity, and a set of the velocity combination to be used in multimodal trajectory prediction is obtained. First, the steps set S is defined as follow:

(6) $$S={\left\{s_{j}\right\}}_{j=1}^{m},\;\;\;\;\;\;\mathrm{where}\;m\mathrm{is}\mathrm{the}\mathrm{number}\mathrm{of}\mathrm{predicted}timesteps.$$

As mentioned in Section 3.1.2, our model predicts velocity and yaw after 10, 20, 30, and 40 timesteps. So m=4 in Equation (6).

Our model predicts logits in the $n_{vel}=25$ velocity classes. The logits of velocity classes $L_{vel}^{s_{j}}$ are defined as follow:

(7) $$L_{vel}^{s_{j}}={\left\{l_{i}^{s_{j}}\right\}}_{i=1}^{n_{vel}},\;\;\;\forall s_{j}\in S,\;\;\;\;\;\;\mathrm{where}\;n_{vel}\;\mathrm{is}\mathrm{the}\mathrm{total}\mathrm{number}\mathrm{of}\mathrm{velocity}classes.$$

For each timestep in the steps set S, we quantify the aleatoric uncertainty from velocity prediction. The maximum value among these quantified aleatoric uncertainties is chosen as the aleatoric uncertainty in this trajectory. Aleatoric uncertainty of velocity $\epsilon_{vel}$ is defined as follow:

(8) $$\epsilon_{vel}=\underset{j=1,\ldots ,m}{\max}\epsilon_{vel}^{s_{j}}.$$

As explained in Section 3.2.3, the aleatoric uncertainty can be quantified by the softmax entropy, and since there are 25 classes in total for velocity, the softmax entropy ranges from 0 to 3.22. Empirically, in our velocity prediction task, softmax entropy usually ranges from 0 to 70% of the maximum. Therefore, we represent the aleatoric uncertainty by normalizing softmax entropy to a value between 0 and 1 in that range.

Based on the aleatoric uncertainty of velocity $\epsilon_{vel}$, the number of the multimode in the longitudinal aspect $K_{long}$ is defined as follow:

(9) $$K_{long}=1+\left\lfloor \alpha_{long}\cdot \epsilon_{vel}\right\rfloor ,\;\;\;\;\;\mathrm{where}\;\alpha_{long}\mathrm{is}\mathrm{the}hyperparameter.$$

The normalized aleatoric uncertainty $\epsilon_{vel}$ is multiplied by the coefficient $\alpha_{long}$. In our work, we set $\alpha_{long}=2.9$ so that $K_{long}$ has one of the values 1, 2, or 3 depending on the aleatoric uncertainty. That is, the more uncertain the velocity prediction is, the more possible cases will be considered in trajectory prediction. If increasing the maximum number of the multimode is needed, increase the $\alpha_{long}$ can be an option.

Among the predicted logits, logits with high confidence are selected as the candidates set. The candidates set of velocity classes $C_{vel}^{s_{j}}$ is defined as follow:

(10) $$C_{vel}^{s_{j}}={Top}_{K_{long}}\left(L_{vel}^{s_{j}}\right),\;\;\;\;\;\;\mathrm{where}\;C_{vel}^{s_{j}}\mathrm{is}\mathrm{ordered}\mathrm{by}\mathrm{descending}confidence.$$

${Top}_{K_{long}}()$ is a filter function that selects only the top $K_{long}$ logits with high confidence. The candidates set contains logits of classes divided according to equal-width binning.

By a convert function ${Convert}_{vel}()$, logits are converted into velocities by an inverse transformation based on the binning rule. The velocities set $V^{s_{j}}$ is defined as follow:

(11) $$V^{s_{j}}=\left\{v_{i}^{s_{j}}|v_{i}^{s_{j}}={Convert}_{vel}\left(l_{i}^{s_{j}}\right),l_{i}^{s_{j}}\in C_{vel}^{s_{j}},i\in \left\{1,\ldots ,K_{long}\right\}\right\},\;\forall s_{j}\in S,\;V^{s_{j}}\subset R^{+}.$$

To predict the multimodal trajectory, the top $K_{long}$ most likely velocities in the velocities set $V^{s_{j}}$ at each timestep $s_{j}$ are combined. The velocity combinations set $V_{combo}$ is defined as follows:

(12) $$V_{combo}=\left\{v_{k}|v_{k}=CartesianProduct\left(V^{s_{1}},V^{s_{2}},\ldots ,V^{s_{m}}\right),\;\;\;k\in \left\{1,\ldots ,\;{\left(K_{long}\right)}^{m}\right\}\right\}.$$

For example, if the number of multimode $K_{long}=3$ because the aleatoric uncertainty $\epsilon_{vel}$ was high, the velocity combinations set $V_{combo}$ contains $3^{4}=81$ combinations.

Through the following series of equations, the number of the multimode in the lateral aspect is determined by reflecting the quantified aleatoric uncertainty of yaw, and a set of the yaw combination to be used in the multimodal trajectory prediction is obtained. The method is the same as that for obtaining the velocity combinations set introduced earlier. Our model predicts logits in the $n_{yaw}=71$ yaw classes. The logits of yaw classes $L_{yaw}^{s_{j}}$ are defined as follows:

(13) $$L_{yaw}^{s_{j}}={\left\{l_{i}^{s_{j}}\right\}}_{i=1}^{n_{yaw}},\;\;\;\forall s_{j}\in S,\;\;\;\;\;\;\mathrm{where}\;n_{yaw}\;\mathrm{is}\mathrm{the}\mathrm{total}\mathrm{number}\mathrm{yaw}classes.$$

For each timestep in the steps set S, we quantify the aleatoric uncertainty from yaw prediction. The maximum value among these quantified aleatoric uncertainties is chosen as the aleatoric uncertainty in this trajectory. The aleatoric uncertainty of yaw $\epsilon_{yaw}$ is defined as follows:

(14) $$\epsilon_{yaw}=\underset{j=1,\ldots ,m}{\max}\epsilon_{yaw}^{s_{j}}.$$

There are 71 classes in total for yaw, and the softmax entropy representing aleatoric uncertainty ranges from 0 to 4.28. Like the velocity prediction task, the softmax entropy of yaw prediction empirically ranges from 0 to 70% of the maximum. Therefore, we represent the aleatoric uncertainty by normalizing the softmax entropy to a value between 0 and 1 in that range.

Based on the aleatoric uncertainty of velocity $\epsilon_{yaw}$, the number of the multimode in the longitudinal aspect $K_{lat}$ is defined as follows:

(15) $$K_{lat}=1+\left\lfloor \alpha_{lat}\cdot \epsilon_{yaw}\right\rfloor ,\;\;\;\;\;\;\mathrm{where}\alpha_{lat}\;\mathrm{is}\mathrm{the}hyperparameter.$$

In our work, we set the coefficient $\alpha_{lat}=2.9$ so that $K_{lat}$ has one of the values 1, 2, or 3 depending on the aleatoric uncertainty.

Among the predicted logits, logits with high confidence are selected as the candidates set. The candidates set of yaw classes $C_{yaw}^{s_{j}}$ is defined as follows:

(16) $$C_{yaw}^{s_{j}}={Top}_{K_{lat}}\left(L_{yaw}^{s_{j}}\right),\;\;\;\;\;\;\mathrm{where}\;C_{yaw}^{s_{j}}\;\mathrm{is}\mathrm{ordered}\mathrm{by}\mathrm{descending}confidence.$$

${Top}_{K_{lat}}$ is a filter function that selects only the top $K_{lat}$ logits with high confidence. The candidates set contains logits of classes divided according to equal-width binning.

By a convert function ${Convert}_{yaw}()$, logits are converted into yaws by an inverse transformation based on the binning rule. The yaws set $\Psi^{s_{j}}$ is defined as follows:

(17) $$\Psi^{s_{j}}=\left\{\psi_{i}^{s_{j}}|\psi_{i}^{s_{j}}={Convert}_{yaw}\left(l_{i}^{s_{j}}\right),l_{i}^{s_{j}}\in C_{yaw}^{s_{j}},i\in \left\{1,\ldots ,K_{lat}\right\}\right\},\;\forall s_{j}\in S,\;\Psi^{s_{j}}\subset \left[0\right.,\left.2\pi \right).$$

To predict the multimodal trajectory, the top $K_{lat}$ most likely yaws in the yaws set $\Psi^{s_{j}}$ at each timestep $s_{j}$ are combined. The yaw combinations set $\Psi_{combo}$ is defined as follows:

(18) $$\Psi_{combo}=\left\{\psi_{k}|\psi_{k}=CartesianProduct\left(\Psi^{s_{1}},\Psi^{s_{2}},\ldots ,\Psi^{s_{m}}\right),k\in \left\{1,\ldots ,\;{\left(K_{lat}\right)}^{m}\right\}\right\}.$$

Through the following series of equations, a set of multimodal trajectories is predicted by reflecting the velocity combinations set and the yaw combinations set obtained above. Using the predicted velocity and yaw, we predict the trajectory by the Euler method in the 2-DOF model. To obtain a denser trajectory, the velocity and yaw are interpolated between the current, after 10, 20, 30, and 40 timesteps. In short, our method predicts a trajectory by calculating a series of positions in a total of 10 timesteps with an interval Δt of 5 timesteps (0.25 s).

Each trajectory that makes up the multimodal trajectories is predicted as follows. The current position $p^{0}$, current velocity $v^{0}$, and current yaw $\psi^{0}$ given in the current timestep are actual values, not predicted values. The trajectory predicted in the first interval using these values is unimodal, so the prediction for the first future position $p^{1}$ is as follows:

(19) $$p^{1}=p^{0}+\left(\begin{array}{c} \cos\left(\psi^{0}\right)\\ \sin\left(\psi^{0}\right) \end{array}\right)\cdot v^{0}\cdot \Delta t.$$

After the first interval, even-numbered future positions are predicted using the velocity and yaw interpolated by a linear interpolation function Interp(). The prediction for second future position $p^{2}$ is as follows:

(20) $$p^{2}=p^{1}+\left(\begin{array}{c} \cos\left(Interp\left(\psi_{combo}^{s_{1}},\psi^{0}\right)\right)\\ \sin\left(Interp\left(\psi_{combo}^{s_{1}},\psi^{0}\right)\right) \end{array}\right)\cdot Interp\left(v_{combo}^{s_{1}},v_{0}\right)\cdot \Delta t,\;v_{combo}^{s_{1}}\in v_{k},\;\;\;\;\;\psi_{combo}^{s_{1}}\in \psi_{k}.$$

Subsequent odd-numbered future positions are predicted by directly using velocity from the velocity combinations set and yaw from the yaw combinations set as follows:

(21) $$p^{h}=p^{h-1}+\left(\begin{array}{c} \cos\left(\psi_{combo}^{s_{j}}\right)\\ \sin\left(\psi_{combo}^{s_{j}}\right) \end{array}\right)\cdot v_{combo}^{s_{j}}\cdot \Delta t,\;h\in \left\{3,5,7,9\right\},\;\;\;\;\;j=\frac{1}{2}\left(h-1\right),\;\;v_{combo}^{s_{j}}\in v_{k},\;\;\;\;\;\psi_{combo}^{s_{j}}\in \psi_{k}.$$

On the other hand, as explained earlier, subsequent even-numbered future positions are predicted by using interpolated velocity and yaw as follows:

(22) $$p^{h}=p^{h-1}+\left(\begin{array}{c} \cos\left(Interp\left(\psi_{combo}^{s_{j}},\psi_{combo}^{s_{j-1}}\right)\right)\\ \sin\left(Interp\left(\psi_{combo}^{s_{j}},\psi_{combo}^{s_{j-1}}\right)\right) \end{array}\right)\cdot Interp\left(v_{combo}^{s_{j}},v_{combo}^{s_{j-1}}\right)\cdot \Delta t,\;h\in \left\{4,6,8\right\},\;\;\;\;\;j=\frac{1}{2}h,\;\;\;\;\;v_{combo}^{s_{j}}\in V_{combo},\;\;\;\;\;\psi_{combo}^{s_{j}}\in \Psi_{combo}.$$

As a result, each trajectory τ that constitutes a multimodal trajectories set is defined as follows:

(23) $$\tau_{\left(v_{k},\psi_{k}\right)}=\left\{p^{h}|h=0,\ldots ,9\right\},\;\;\;\;\;v_{k}\in V_{combo},\;\;\;\;\;\psi_{k}\in \Psi_{combo}.$$

Ultimately, the multimodal trajectories set at time t is $T\left(t\right)$ and is defined as follows:

(24) $$T\left(t\right)=\underset{v_{k}\in V_{combo},\psi_{k}\in \Psi_{combo}}{⋃}\tau_{\left(v_{k},\psi_{k}\right)}.$$

To briefly explain our trajectory expansion method, if the epistemic uncertainty for yaw is high, the predicted trajectory is expanded in the lateral direction, and if the epistemic uncertainty for velocity is high, the predicted trajectory is expanded in the longitudinal direction. As in the case of aleatoric uncertainty quantification, for each timestep in the steps set S, we quantify the epistemic uncertainty from the velocity prediction. The maximum value among these quantified epistemic uncertainties is chosen as the epistemic uncertainty in this trajectory. The epistemic uncertainty of velocity $\theta_{vel}$ is defined as follows:

(25) $$\theta_{vel}=\underset{j=1,\ldots ,m}{\max}\theta_{vel}^{s_{j}}.$$

By reflecting the epistemic uncertainty of velocity $\theta_{vel}$, the expanded trajectory in the longitudinal direction is calculated as follows:

(26) $$\begin{array}{l} P_{long}\left(x|T_{long},\theta_{vel}\right)\\ =\{\begin{array}{cc} \frac{1}{2\cdot \beta_{long}\cdot \theta_{vel}} & \mathrm{for}\;x\in \left[T_{long}\left(t\right)-\beta_{long}\cdot \theta_{vel},\;\;\;\;\;T_{long}\left(t\right)+\beta_{long}\cdot \theta_{vel}\right]\\ 0 & \;\;\;\;\mathrm{otherwise} \end{array} \end{array},\;\mathrm{where}\;\beta_{long}\mathrm{is}\mathrm{the}hyperparameter.$$

The formula is in a form of a probability density function of a uniform distribution, which expands the predicted trajectory $T_{long}$ in the longitudinal direction according to the epistemic uncertainty $\theta_{vel}$ and coefficient $\beta_{long}$. If we want to make vehicles with high epistemic uncertainty less trustworthy and thus respond more conservatively to their future motions, we can increase the value of coefficient $\beta_{long}$.

For each timestep in the steps set S, we quantify the epistemic uncertainty from yaw prediction. The maximum value among these quantified epistemic uncertainties is chosen as the epistemic uncertainty in this trajectory. The epistemic uncertainty of yaw $\theta_{yaw}$ is defined as follows:

(27) $$\theta_{yaw}=\underset{j=1,\ldots ,m}{\max}\theta_{yaw}^{s_{j}}.$$

In the same way as described above, by reflecting the epistemic uncertainty of yaw $\theta_{yaw}$, the expanded trajectory in the lateral direction is calculated as follows:

(28) $$\begin{array}{l} P_{lat}\left(x|T_{lat},\theta_{yaw}\right)\\ =\{\begin{array}{cc} \frac{1}{2\cdot \beta_{lat}\cdot \theta_{yaw}} & \mathrm{for}\;x\in \left[T_{lat}\left(t\right)-\beta_{lat}\cdot \theta_{yaw},\;\;\;\;\;T_{lat}\left(t\right)+\beta_{lat}\cdot \theta_{yaw}\right]\\ 0 & \mathrm{otherwise}, \end{array}\\ \mathrm{where}\;\beta_{lat}\mathrm{is}\mathrm{the}hyperparameter. \end{array}$$

## 4. Experiments

### 4.1. Evaluation of Decomposed Trajectory Prediction Task

In Section 3.1, we described an approach that predicts velocity and yaw classes with equal-width binning applied and then uses the Euler method in a 2-DOF model based on predicted velocity and yaw to predict the future trajectory. As detailed in that section, our model for predicting velocity and yaw has a ResNet-based architecture.

As mentioned in Section 3.2, we applied three state-of-the-art uncertainty quantification methods to our model: Monte Carlo Dropout, Deep Ensembles, and deterministic single forward pass. However, uncertainty quantification methods impose additional resource and computational burdens on edge platforms of autonomous vehicles which have limited memory and computing power. For real-time responsiveness to ensure the safety of vehicles, the computational burden is an important issue in autonomous driving systems. It is so critical that it can determine the feasibility of adopting uncertainty quantification methods in trajectory prediction tasks.

In this section, we compare three uncertainty quantification methods applied to our trajectory prediction model with metrics that evaluate both the performance and efficiency of the methods. The implementation of each method is described in Section 3.2.2. We measure the accuracy of velocity and yaw predictions, which corresponds to the metric for the performance of the predictor models, and also measure the number of trainable parameters, the number of floating-point operations (FLOPs), and the runtime including both prediction and uncertainty quantification processes, which correspond to the metrics for the efficiency of the methods.

Specifications of our hardware used to measure the runtime are a single Intel Xeon Gold 6140 CPU with 18 cores and a single Nvidia GeForce RTX 2080 Ti GPU with 13.45 TFLOPS cores and 11 GB VRAM. Since the platforms mounted on autonomous vehicles do not have high-end workstation-level specifications, we selected a moderate level of hardware in our evaluation.

In the following series of tables showing the evaluation results, MCD stands for Monte Carlo Dropout, DE stands for Deep Ensemble, and DSFP stands for deterministic single forward pass. The upward arrow indicates that the higher the value, the better, and the downward arrow indicates that the lower the value, the better. The bold number indicates the best value, and the underlined number indicates the second-best value.

As shown in Table 3, Deep Ensemble shows the highest accuracy among the three methods. The high accuracy of Deep Ensembles comes from the structural advantage of using multiple models [12,13,65]. However, the accuracy gain of Deep Ensembles shows a small difference of about 1%p compared to the deterministic single forward pass method.

Unfortunately, since the ensemble contains multiple models, the number of trainable parameters and FLOPs increase proportionally to the number of models. It requires much more memory than Monte Carlo Dropout and deterministic single forward pass using a single model. The large memory demand of Deep Ensembles may not be feasible for autonomous driving systems with limited amounts of memory.

In addition, the runtime per inference of Deep Ensembles that require multiple models and Monte Carlo Dropout that requires dozens of samplings were 3.26 to 21.62 times higher than that of the deterministic single forward pass. We measured the time required for prediction and uncertainty quantification of ten vehicles as the runtime per inference. The runtime is the average time measured per timestep in 15 driving sequences that are 5000 timesteps long. Heuristically, it is highly recommended that autonomous driving systems operate at a minimum rate of 20 Hz to meet the real-time responsiveness that ensures safety of the vehicle, and our evaluation shows that only the deterministic single forward pass method can achieve this rate.

Table 4 shows separate accuracy results after 10, 20, 30, and 40 timesteps for reference.

### 4.2. Evaluation of Uncertainty Quantification

In many studies addressing the uncertainty of deterministic models, AUROC (Area Under the Receiver Operating Characteristic) and ECE (Expected Calibration Error) are used as metrics to measure the quality of uncertainty quantification [66]. However, for deterministic uncertainty methods that quantify uncertainty in the form of a distance or density-related score, the ECE, which measures the calibration of probabilities, is not a suitable metric because there is no direct association between the uncertainty score and the probabilistic prediction [63,67].

In our evaluation, we utilize two types of AUROC metrics used in state-of-the-art studies on uncertainty quantification to evaluate the uncertainty quantification methods. The typical AUROC metric used in evaluation of binary classification models is calculated using binary labels representing positive/negative classes and predicted probabilities. However, the AUROC metric used in evaluating uncertainty quantification methods is different.

The first type of AUROC, which we named AUROC-AU, is the AUROC between a binary label indicating whether a prediction is wrong or not and the aleatoric uncertainty expressed as a score. AUROC-AU indicates how well the model identifies cases where its prediction is likely to be wrong due to environmental randomness, suggesting that aleatoric uncertainty effectively reflects prediction failure. An AUROC-AU value close to 100 indicates that the model assigns high aleatoric uncertainty to wrong predictions. On the other hand, an AUROC-AU value close to 50 indicates that there is little correlation between aleatoric uncertainty and prediction failure.

The second type of AUROC, which we call AUROC-EU, is the AUROC between a binary label indicating whether a prediction is wrong or not and the epistemic uncertainty expressed as a score. AUROC-EU indicates how well the model recognizes the limitations of its own knowledge, suggesting that epistemic uncertainty effectively reflects the detection of OOD due to a lack of knowledge. AUROC-EU values close to 100 indicate that the model assigns high epistemic uncertainty to OOD samples. On the other hand, values close to 50 indicate that there is little correlation between epistemic uncertainty and OOD detection. The method of quantifying aleatoric and epistemic uncertainty in our experiments follows the description in Section 3.2.3.

To evaluate uncertainty quantification methods with the AUROC-AU metric, we performed predictions on the test dataset and assigned binary labels of 1 if the prediction was correct and 0 if it was incorrect. Then, we paired the quantified aleatoric uncertainty with the binary label for the corresponding prediction.

Table 5 shows that the deterministic single forward pass approach, which quantifies aleatoric uncertainty with softmax entropy, quantifies aleatoric uncertainty with the best quality in the trajectory prediction problem where environmental randomness that can lead to incorrect predictions is inherent.

In Section 2.1.1, we mentioned that aleatoric uncertainty can quantify the ambiguity of data due to randomness, and in Section 3.3.1, we explained that trajectory prediction is a multimodal problem due to the randomness inherent in the driving environment. The high AUROC-AU in our evaluation supports the validity of our uncertainty-aware multimodal trajectory prediction method that utilizes aleatoric uncertainty quantifying environmental randomness in driving situations, as proposed in Section 3.3.2.

Table 6 shows separate AUROC-AU after 10, 20, 30, and 40 timesteps for reference.

To evaluate the uncertainty quantification methods with the AUROC-EU metric, we performed predictions on the test dataset including both ID and OOD data and assigned binary labels of 1 if the data sample was from the OOD environment and 0 if it was from the ID environment. As explained in Section 3.1.3, the OOD sample is taken from the Town06 map, which is a highway environment, and the ID sample is taken from the Town03 map, which is a downtown environment. Then, we paired the quantified epistemic uncertainty with the binary label for the corresponding prediction.

Table 7 shows that the deterministic single forward pass approach, which quantifies epistemic uncertainty by measuring the marginal likelihood of the feature under the feature space, quantifies epistemic uncertainty with the best quality in the trajectory prediction task. The OOD detection capability of epistemic uncertainty quantified by Monte Carlo Dropout is found to be incompetent. The typical AUROC metric is considered to range between 50 and 100, but the actual possible range is between 0 and 100. A value lower than 50 means that the correlation between the binary label and score is inversely proportional.

Based on the high AUROC-EU value close to 100, it is possible to expand the predicted trajectory only in OOD driving situations by utilizing the quantified epistemic uncertainty, as proposed in Section 3.3.2.

Table 8 shows separate AUROC-EU results after 10, 20, 30, and 40 timesteps for reference.

According to Table 8, Deep Ensembles show worse OOD detection capability for yaw predictions at distant timesteps than the deterministic single forward pass method.

The capability of Deep Ensembles in epistemic uncertainty quantification is not significantly inferior to that of the deterministic single forward pass method. However, as shown in Table 2 and Table 3, the Deep Ensemble requires eight models, which incurs larger computational burdens than a single model. However, we cannot downsize the number of models that make up the ensemble. Table 9 shows that the capability of Deep Ensembles to quantify epistemic uncertainty becomes unstable when the number of models is small. This is because as the number of models decreases, it becomes more difficult to robustly measure the variance of predictions.

Based on the evaluation results so far, we conclude that the deterministic single forward pass method is the most feasible among the three state-of-the-art uncertainty quantification methods in the trajectory prediction task, as it has the lowest computational burden while maintaining good prediction accuracy. Deep Ensembles that require high memory and computing power may not be feasible on the edge platform of autonomous vehicles with limited hardware specifications. To achieve real-time responsiveness, it should also meet the low runtime requirement, but other uncertainty quantification methods failed to do so.

And as a result of evaluating the uncertainty quantification methods with the AUROC-AU and AUROC-EU metrics, we come to conclusion that the deterministic single forward pass method, which always show better values than other methods, is the most suitable for applying uncertainty-aware multimodal trajectory prediction.

### 4.3. Evaluation of Uncertainty-Aware Multimodal Trajectory Prediction

In this section, we demonstrate the effectiveness of our uncertainty-aware multimodal trajectory prediction (UAMTP) method proposed in Section 3.3, which adopts the deterministic single forward pass method for the uncertainty quantification. We evaluate the multimodal trajectory prediction task with two metrics that are the default in this field [68]. One metric is the minFDE (Minimum Final Displacement Error), which measures the closest distance between the final coordinates of multimodal trajectories and the final coordinate of a ground truth trajectory. The other metric is the miss rate, which indicates the proportion of minFDEs that fail to satisfy the threshold among all result samples. The threshold for the miss rate is set to 1 m, which is suggested in previous studies, and 1.5 m, which is one-third of the length of typical passenger vehicles [69].

Table 10 shows how much our UAMTP method predicts trajectories more accurately than other trajectory prediction methods in driving situations where the target vehicle turns. We prepared three other methods to compare with our method. First, the baseline is a unimodal trajectory prediction method that predicts the most-likely trajectory by a single model. Second, Deep Ensembles (DEs)-based unimodal trajectory prediction is a method that predicts the most-likely trajectory by averaging the predictions of eight models. Third, Deep Ensembles (DEs)-based multimodal trajectory prediction method outputs the predictions of eight models as a set of multimodal trajectories [39,40,69]. Since the eight models that make up the ensemble are trained with differently sampled datasets, generalization can be expected, and various predictions can be output in driving situations where randomness exists [65].

In Section 3.3.2, we explained that our UAMTP method can perform aleatoric uncertainty-aware multimodal trajectory prediction and epistemic uncertainty-aware trajectory expansion. However, the Deep Ensembles-based multimodal trajectory prediction method cannot adopt trajectory expansion because it does not utilize uncertainty. For a fair comparison, in all evaluations in Section 4.3, our UAMTP only performed multimodal trajectory prediction without trajectory expansion.

In this evaluation, data are sampled in vehicle-turning situations, which can be potentially more dangerous than vehicles simply going straight ahead. The Town03 map we used for testing is an urban environment with multiple crossroads and a roundabout, which causes environmental randomness due to the road structure with multiple branching, as mentioned in Section 2.3.2 and Section 3.3.1. Our method of performing multimodal trajectory prediction by quantifying aleatoric uncertainty, which measures the randomness of the driving environment in the data, is effective in reducing prediction errors and miss rates.

The Argoverse benchmark performs multimodal trajectory prediction with up to six predictions, but in our evaluation, the DE-based multimodal prediction, which is our comparison target, leverages more predictions, eight, and thus can be expected to predict trajectories more broadly [69]. Nevertheless, DE-based multimodal prediction does not outperform our UAMTP method.

In addition, ablation studies in Table 11 demonstrate that our UAMTP method is effective in the longitudinal and lateral directions, respectively. For the ablation studies, we applied uncertainty-aware multimodal prediction only in the longitudinal direction depending on the aleatoric uncertainty of velocity or in the lateral direction depending on the aleatoric uncertainty of yaw and applied unimodal prediction in the remaining directions.

Table 10 shows the results evaluated only for situations where the vehicle turns, while Table 12 shows the results evaluated for all driving situations where vehicles are turning, moving straight, and stopped. Since it is easy to predict the stationary state of the vehicle, the error and miss rates are lower than in Table 10. Under this evaluation condition, our UAMTP method shows similar or slightly better results than DE-based multimodal prediction. Our method falls behind the DE-based multimodal prediction method by 0.7%p in the miss rate metric at the 1.0 m threshold but outperforms it in other metrics.

As mentioned in Section 4.1, the time efficiency of prediction models is very important in autonomous driving systems for applicability and safety assurance. To verify this, we also evaluate the runtime required for multimodal trajectory prediction. The runtime is the time required to predict five surrounding vehicles per timestep. Five vehicles are enough to completely surround an ego vehicle. For a fair comparison, we impose the computational load on UAMTP by including at least one uncertain vehicle per timestep in each driving sequence. The UAMTP can be exposed to cases even where all five cars are uncertain. Table 13 shows the runtime measured on DE-based multimodal trajectory prediction and our UAMTP. The reported runtime is the average time per timestep across 15 driving sequences, each consisting of 5000 timesteps. Measurement results show that our UAMTP achieves 1.11 times faster runtime than DE-based multimodal trajectory prediction. This result stems from UAMTP’s approach, performing multimodal trajectory prediction only when the quantified aleatoric uncertainty is high.

So far, we have explained the results of evaluating UAMTP in the driving situation where environmental randomness exists due to road structures such as crossroads and roundabouts. In this additional experiment, we evaluate whether UAMTP can contribute to the safety of the vehicle in the situation where aleatoric uncertainty due to the randomness of the agent’s intention exists. We placed a target vehicle in the lane next to the ego vehicle in a two-lane roundabout and made the target vehicle drive slightly ahead of the ego vehicle. In the scenario we constructed, the target vehicle suddenly crosses the ego vehicle’s lane without any warning and exits the roundabout. In this scenario, we evaluate how much more time our UAMTP can provide autonomous driving systems to react to potential collisions, compared to the baseline unimodal trajectory prediction.

**Figure 4:**
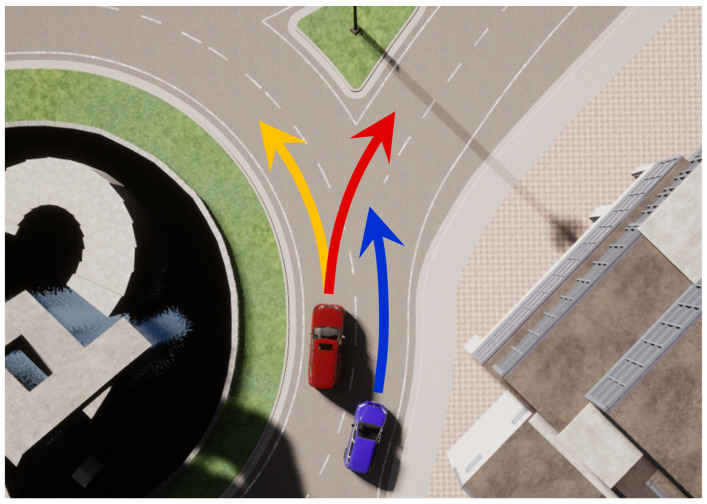
Description of the roundabout scenario. The blue vehicle is the ego vehicle trying to pass through the roundabout following the blue arrow, and the red vehicle is the vehicle trying to exit the roundabout by suddenly cutting in front of the ego vehicle. The yellow arrow is the trajectory incorrectly predicted by the unimodal trajectory predictor right until the red vehicle starts to actively exit the roundabout by leaving its lane.

Figure 4 depicts the roundabout scenario. The time to react that we define is the time that the ego vehicle, the blue vehicle, can take to react from the moment it recognizes that its path intersects the predicted trajectory of the neighboring red vehicle until a collision occurs if the ego vehicle takes no action. In Figure 4, the yellow arrow is the only trajectory for the red vehicle predicted by the unimodal trajectory predictor model right until the vehicle starts to actively exit the roundabout by leaving its lane. It is because the unimodal trajectory predictor predicts a single trajectory that seems most likely based on the learned data.

However, the aleatoric uncertainty increases due to the randomness of the intention that other agents may escape the roundabout near the exit of the roundabout. As the aleatoric uncertainty increases, our UAMTP model predicts the yellow and red arrows together as the future trajectories of the red vehicle more proactively than the unimodal trajectory predictor. This is also depicted in Figure 3 with the dynamic graph. Table 14 shows the experiment results in this scenario. Our UAMTP utilizing aleatoric uncertainty can secure a time to react that is 1.28 times longer than the baseline. Thanks to the longer time to react, the autonomous driving system of the ego vehicle has more room to take safer actions.

## 5. Discussion and Conclusions

In this study, we have extensively reviewed previous studies on various uncertainty quantification methods. Considering the characteristics of edge platforms of autonomous vehicles with limited memory and computing power, we experimentally identified uncertainty quantification methods that are applicable to trajectory prediction tasks of autonomous driving systems. Compared to the state-of-the-art Deep Ensembles with 8 models, the deterministic single forward pass method had only 1/8 the number of trainable parameters and FLOPs and showed only 1/3 the runtime. We concluded that the deterministic single forward pass method that can quickly quantify uncertainty in a memory-efficient manner without compromising prediction performance is suitable for applying uncertainty quantification to autonomous driving while satisfying the real-time responsiveness of systems. We believe this lightweight method also has the potential to be adopted for platforms that have stricter hardware limitations than autonomous vehicles, such as delivery robots.

Furthermore, we showed that the multimodality problem of the trajectory prediction task due to the environmental randomness inherent in the driving environment can be quantified as aleatoric uncertainty. The AUROC-AU metric demonstrated that aleatoric uncertainty can quantify randomness in the driving environment, which can lead to incorrect trajectory predictions. To address the multimodal trajectory prediction suffering from randomness, we proposed the uncertainty-aware multimodal trajectory prediction (UAMTP), which integrates uncertainty quantification into trajectory prediction. For our UAMTP method, we adopted the deterministic single forward pass, which recorded the highest AUROC-AU and AUROC-EU metrics compared to other state-of-the-art methods, in addition to the efficiency evaluated above. To the best of our knowledge, our UAMTP is the first study to integrate quantified uncertainty into the prediction of multimodal trajectories. This approach, which performs multimodal trajectory prediction while quantifying uncertainty, has the potential to improve the safety of autonomous vehicles. The evaluation results with minFDE and miss rate metrics support this. The UAMTP method leveraging a single inference from a single model outperforms the Deep Ensembles-based multimodal trajectory prediction using eight models. The advantage of UAMTP is particularly evident when predicting turning vehicles. The minFDE is reduced by 13%, and the miss rate is reduced by 24% when the threshold is 1.5 m. In terms of runtime, UAMTP is also more competitive than the Deep Ensembles-based method. UAMTP’s runtime is 1.11 times faster than the Deep Ensemble’s. Additionally, the evaluation result that the time to react of UAMTP was increased by 1.28 times compared to the unimodal prediction in the roundabout scenario shows that UAMTP can be effective in ensuring the safety of autonomous vehicles by providing a time margin for decision-making when integrated with the decision-making algorithm of the autonomous driving system.

Given that our UAMTP is the first attempt to integrate uncertainty quantification and multimodal trajectory prediction, in this work, we focus on demonstrating efficient performance improvement in trajectory prediction. As for future works, the utilization of epistemic uncertainty can be expanded to not only the trajectory expansion proposed in this work but to a self-improvement of the model by collecting OOD samples from driving situations. When testing a learned model, if driving situations are found where the epistemic uncertainty of the prediction is high, the model can be updated by collecting only these uncertain situations and additionally learning them.

As mentioned in Section 2.3.2, there is room for improvement in prediction accuracy if our UAMTP is combined with a part that explicitly models uncertainty related to driving style or driver intention. However, careful consideration is needed because introducing a new uncertainty source may lead to additional problems.

Ultimately, future work could extend the framework by integrating uncertainty-aware multimodal trajectory prediction with the planning subsystem of an autonomous driving system. If the intersection between predicted trajectories is modeled as a potential risk and used as a reward, multi-agent training based on reinforcement learning can be one of the main options. Moreover, further development may include real-world deployment on autonomous vehicles to evaluate performance under non-simulated conditions. These advancements will be essential for achieving a higher level of reliability and robustness in autonomous driving systems, moving closer to the deployment of fully autonomous vehicles in unrestricted environments.

## Figures and Tables

**Table 1:** Data components constituting a state vector of a vehicle.

Attribute	Unit
Position x, y	meter
Yaw	degree
Velocity x, y	m/s
Yaw rate	degree/s
Acceleration x, y	m/s^2^
Is the vehicle at stop line	true or false
Traffic light affecting the vehicle	red or yellow or green

**Table 2:** Summary of key specifications of state-of-the-art uncertainty quantification methods covered in our evaluation. In the following series of tables in this paper, the upward arrow indicates that the higher the value, the better, and the downward arrow indicates that the lower the value, the better. The bold number indicates the best value, and the underlined number indicates the second-best value. The deterministic single forward pass method, which requires only a single inference from a single model, suffers less from computational burden than other methods.

	# of Models (↓)	# of Inferences (↓)	Computational Burden (↓)
Monte Carlo Dropout	1	16	High (Monte Carlo sampling)
Deep Ensembles	8	1 per model	High (Bootstrap aggregating)
Deterministic Single Forward Pass	**1**	**1**	**Moderate** **(Distance/Density calculation)**

**Table 3:** Evaluation results of the performance and efficiency of predictor models reflecting three state-of-the-art uncertainty quantification methods.

	Accuracy (↑)		# of Trainable Params (↓)	# of FLOPs (↓)	Runtime per Inference (↓)
	Velocity	Yaw			
MCD	79.8890 ± 0.1442	94.3683 ± 0.0799	**4,384,808**	**991,974,024**	0.735 s (21.62×)
DE	**81.7350 ± 0.1890**	**95.0141 ± 0.0786**	35,078,464	7,935,792,192	0.111 s (3.26×)
**DSFP**	80.8642 ± 0.2279	94.3762 ± 0.0949	**4,384,808**	**991,974,024**	**0.034 s (1×)**

**Table 4:** Detailed evaluation results of the performance of predictor models reflecting three state-of-the-art uncertainty quantification methods. The results show separate accuracy scores for predictions of velocity and yaw after 10, 20, 30, and 40 timesteps, instead of the averaged accuracy independent of timesteps.

	Accuracy (↑)							
	Velocity				Yaw			
	After-10	After-20	After-30	After-40	After-10	After-20	After-30	After-40
MCD	81.7733 ± 0.2923	80.6975 ± 0.2921	79.6937 ± 0.2912	77.3916 ± 0.2781	94.3683 ± 0.0799	96.7710 ± 0.0843	95.5265 ± 0.1328	91.5189 ± 0.2259
DE	**83.2975 ± 0.3482**	**82.4998 ± 0.3817**	**81.3326 ± 0.3678**	**79.8101 ± 0.4114**	**97.2709 ± 0.0811**	**96.0232 ± 0.1208**	**94.3470 ± 0.1576**	**92.4154 ± 0.2301**
**DSFP**	82.2107 ± 0.4408	81.5333 ± 0.4512	80.6762 ± 0.4672	79.0366 ± 0.4636	96.8556 ± 0.1114	95.3846 ± 0.1558	93.8225 ± 0.1929	91.4419 ± 0.2650

**Table 5:** Evaluation results of the aleatoric uncertainty quantification capability of three state-of-the-art methods. The quantified aleatoric uncertainty for velocity prediction and one for yaw prediction are evaluated separately by the AUROC-AU metric.

	AUROC-AU (↑)	
	Aleatoric Uncertainty of Velocity	Aleatoric Uncertainty of Yaw
MCD	84.1455 ± 0.1097	96.7504 ± 0.0392
DE	89.3743 ± 0.1300	97.0878 ± 0.0433
**DSFP**	**89.9730 ± 0.1464**	**97.1380 ± 0.0440**

**Table 6:** Detailed evaluation results of the aleatoric uncertainty quantification capability of three state-of-the-art methods. The quantified aleatoric uncertainty for velocity prediction and one for yaw prediction are evaluated separately by the AUROC-AU metric. The results show separate AUROC-AU for predictions of velocity and yaw after 10, 20, 30, and 40 timesteps, instead of the averaged AUROC-AU independent of timesteps.

	AUROC-AU (↑)							
	Aleatoric Uncertainty of Velocity				Aleatoric Uncertainty of Yaw			
	After-10	After-20	After-30	After-40	After-10	After-20	After-30	After-40
MCD	90.0805 ± 0.1379	87.5751 ± 0.1549	82.3324 ± 0.2579	76.5939 ± 0.2884	97.2907 ± 0.0685	97.1138 ± 0.0813	96.6549 ± 0.0690	95.9420 ± 0.0922
DE	91.1284 ± 0.2332	90.3828 ± 0.2417	89.0760 ± 0.2653	86.9101 ± 0.2955	97.5207 ± 0.0877	97.3861 ± 0.0908	97.0043 ± 0.0794	96.4400 ± 0.0882
**DSFP**	**91.8791 ± 0.2961**	**91.3033 ± 0.2763**	**89.3830 ± 0.2911**	**87.1825 ± 0.3071**	**97.5296 ± 0.0871**	**97.3894 ± 0.0862**	**97.0558 ± 0.0873**	**96.5722 ± 0.0913**

**Table 7:** Evaluation results of the epistemic uncertainty quantification capability of three state-of-the-art methods. The quantified epistemic uncertainty for velocity prediction and yaw prediction are evaluated separately by the AUROC-EU metric.

	AUROC-EU (↑)	
	Epistemic Uncertainty of Velocity	Epistemic Uncertainty of Yaw
MCD	49.1900 ± 0.2354	54.6307 ± 0.2145
DE	98.3863 ± 0.0328	97.0878 ± 0.0756
**DSFP**	**99.8110 ± 0.0074**	**99.8643 ± 0.0051**

**Table 8:** Detailed evaluation results of the epistemic uncertainty quantification capability of three state-of-the-art methods. The quantified epistemic uncertainty for velocity prediction and yaw prediction are evaluated separately by the AUROC-EU metric. The results show separate AUROC-EU values for predictions of velocity and yaw after 10, 20, 30, and 40 timesteps, instead of the averaged AUROC-EU independent of timesteps.

	AUROC-EU (↑)							
	Aleatoric Uncertainty of Velocity				Aleatoric Uncertainty of Yaw			
	After-10	After-20	After-30	After-40	After-10	After-20	After-30	After-40
MCD	46.9658 ± 0.4285	48.2709 ± 0.4711	50.1777 ± 0.5457	51.3455 ± 0.4280	54.3445 ± 0.3983	52.6054 ± 0.4246	55.5397 ± 0.4356	56.0333 ± 0.4558
DE	99.0935 ± 0.0276	99.2409 ± 0.0185	95.5325 ± 0.1262	99.6784 ± 0.0122	99.8101 ± 0.0132	99.8145 ± 0.0107	94.4022 ± 0.2082	94.2220 ± 0.2188
**DSFP**	**99.7687 ± 0.0172**	**99.7652 ± 0.0163**	**99.8488 ± 0.0110**	**99.8613 ± 0.0141**	**99.9485 ± 0.0061**	**99.8839 ± 0.0078**	**99.8055 ± 0.0100**	**99.8192 ± 0.0146**

**Table 9:** Evaluation results of the epistemic uncertainty quantification capability of the Deep Ensembles method according to the number of models. DE-8 is a Deep Ensembles method that includes 8 models used in the evaluation, and DE-4 includes only 4 models for the ablation study. The quantified epistemic uncertainty for velocity prediction and for yaw prediction are evaluated separately by the AUROC-EU metric.

	AUROC-EU (↑)	
	Epistemic Uncertainty of Velocity	Epistemic Uncertainty of Yaw
DE-4	75.9640 ± 0.2449	93.2563 ± 0.0863
DE-8	**98.3863 ± 0.0328**	**97.0622 ± 0.0756**

**Table 10:** Evaluation results of the performance of trajectory prediction methods in driving situations where the target vehicle turns.

	minFDE (↓)	Miss Rate (>1.0 m) (↓)	Miss Rate (>1.5 m) (↓)
Unimodal Prediction (Baseline)	2.138 ± 0.009 m	0.751	0.437
DE-based Unimodal Prediction	2.030 ± 0.008 m	0.718	0.418
DE-based Multimodal Prediction	1.281 ± 0.005 m	0.423	0.222
**UAMTP** **(Ours)**	**1.112 ± 0.005 m**	**0.383**	**0.168**

**Table 11:** Evaluation results of the ablation studies on our uncertainty-aware multimodal trajectory prediction (UAMTP) method in driving situations where the target vehicle turns. Longitudinal-only and lateral-only UAMTP methods are intended for ablation studies.

	minFDE (↓)	Miss Rate (>1.0 m) (↓)	Miss Rate (>1.5 m) (↓)
Unimodal (Baseline)	2.138 ± 0.009 m	0.751	0.437
Longitudinal-only UAMTP (Ablation)	1.615 ± 0.006 m	0.648	0.337
Lateral-only UAMTP (Ablation)	1.682 ± 0.008 m	0.515	0.281
**UAMTP** **(Ours)**	**1.112 ± 0.005 m**	**0.383**	**0.168**

**Table 12:** Evaluation results of the performance of trajectory prediction methods in all driving situations.

	minFDE (↓)	Miss Rate (>1.0 m) (↓)	Miss Rate (>1.5 m) (↓)
Unimodal Prediction (Baseline)	1.085 ± 0.003 m	0.330	0.201
DE-based Unimodal Prediction	1.006 ± 0.003 m	0.310	0.182
DE-based Multimodal Prediction	0.637 ± 0.002 m	**0.170**	0.094
**UAMTP** **(Ours)**	**0.618 ± 0.002 m**	0.177	**0.085**

**Table 13:** Evaluation results of the time efficiency of multimodal trajectory prediction methods.

	Runtime per Prediction (↓)
DE-based Multimodal Prediction	0.091 ± 0.001 s (1.11×)
**UAMTP** **(Ours)**	**0.082 ± 0.002 s (1×)**

**Table 14:** Experiment results for time to react of uncertainty-aware multimodal trajectory prediction (UAMTP) in roundabout scenario.

	Time to React (↑)
Unimodal Prediction (Baseline)	1.49 s (×1)
**UAMTP** **(Ours)**	**1.91** **s** **(×1.28)**

## Data Availability

The function for collecting the data used in this research from the CARLA simulator, as well as the implementation of the method proposed in this article, are available through a GitHub repository: https://github.com/TigerStone93/UAMTP (accessed on 11 November 2024).
